# P-67. Safety of Omadacycline Versus Standard-of-Care Oral Antibiotic Treatment for Bone and Joint Infections: Interim Results from an Open-Label, Non- Inferiority, Randomized Controlled Trial

**DOI:** 10.1093/ofid/ofae631.274

**Published:** 2025-01-29

**Authors:** Amy Y Kang, Guarina Garcia Delgado, Donna Phan Tran, Evelyn A Flores, Vanessa Romo-Kozan, Isabel Payan, Loren G Miller

**Affiliations:** Chapman University / Harbor-UCLA Medical Center, Irvine, California; Lundquist Institute at Harbor-UCLA Medical Center, Claremont, California; Division of Infectious Diseases, the Lundquist Institute at Harbor-UCLA Medical Center, Torrance, CA, Torrance, California; Division of Infectious Diseases, the Lundquist Institute at Harbor-UCLA Medical Center, Torrance, CA, Torrance, California; Lundquist Institute, Raleigh, North Carolina; The Lundquist Institute, COMPTON, California; Lundquist Institute at Harbor-UCLA Medical Center, Claremont, California

## Abstract

**Background:**

The incidence of bone and joint infections (BJIs) continues to increase, and existing oral BJI antibiotics have limitations. Omadacycline may be a potential treatment option for BJI treatment due to activity against doxycycline-resistant *S. aureus* and ESBL-producing Enterobacterales for which there are often no viable oral options. However, the safety of omadacycline in this setting is poorly defined.

**Figure:**
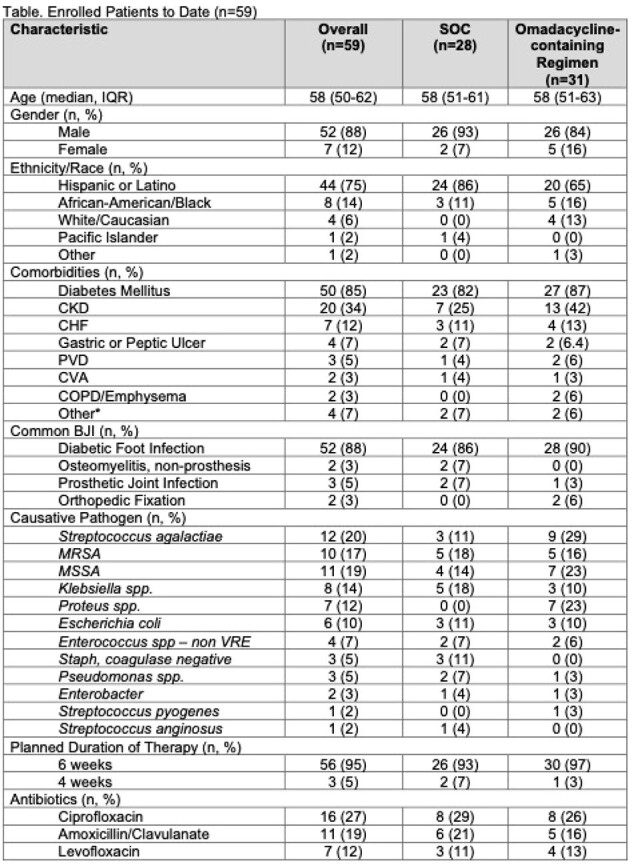

**Methods:**

We performed an open label, randomized, controlled clinical trial of adults with BJIs in the outpatient setting. Participants were randomized to omadacycline-containing regimen (omadacycline 300mg po daily) or Standard-of-Care (SOC) antibiotics. Adjunctive antibiotics were permitted in addition to omadacycline. Safety labs were performed at regular intervals. Herein, we report an interim, descriptive analysis of safety data.

**Figure:**
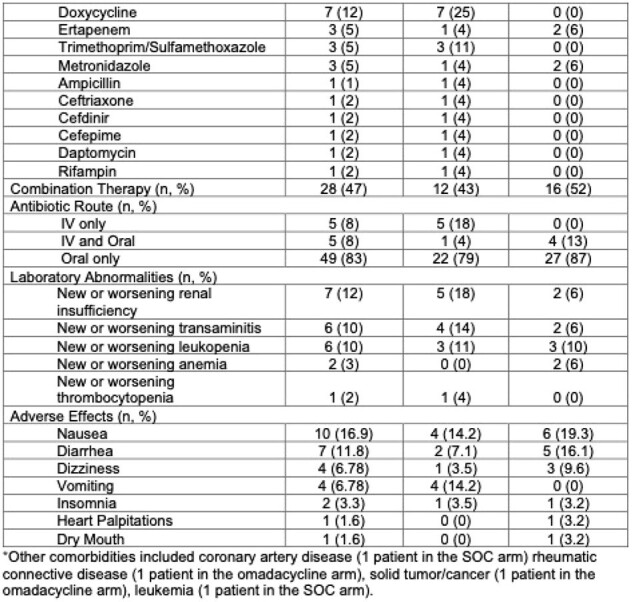

**Results:**

Among the 59 participants enrolled to date, 31 (53%) were randomized to the omadacycline-containing regimen and 28 (47%) to SOC. Median age was 58 years old, 52 (88%) were male, 44 (75%) identified as Hispanic, 8 (14%) as African-American, and 4 (6%) as white. The most common BJIs were diabetic foot infections (52, 88%) and device infections (5, 8%). (Table) Combination therapy was used in 16 (52%) vs. 12 (43%) in the omadacycline-containing vs. SOC arms, and IV only therapy was used in 0 (%) vs. 5 (18%) in the two arms, respectively. Most commonly used SOC regimens included ciprofloxacin (8, 29%), doxycycline (7, 25%), amoxicillin/clavulanate (6, 21%), and levofloxacin (3, 11%). Commonly associated laboratory abnormalities included new or worsening renal insufficiency (2 (6%) in the omadacycline-containing arm vs. 5 (18%) in SOC) and transaminitis (2 (6%) vs. 4 (14%)). Adverse events were similar in the omadacycline-containing vs. SOC arms including nausea (6 (19%) vs. 4 (14%)), vomiting (0 (0%) vs. 4 (14%)), and diarrhea (5 (16%) vs. 2 (7%)). (Table) There was 1 drug-related serious adverse event in the omadacycline-containing arm due to likely hypersensitivity to omadacycline; the event resolved after drug discontinuation.

**Conclusion:**

In this interim analysis of our ongoing trial, the safety of omadacycline for BJI appears to be similar to the SOC. More robust data on long-term safety is warranted.

**Disclosures:**

**Amy Y. Kang, Pharm.D., BCIDP**, Paratek Pharmaceutical: Grant/Research Support **Loren G. Miller, MD MPH**, Armata: Grant/Research Support|Contrafect: Grant/Research Support|GSK: Grant/Research Support|Merck: Grant/Research Support|Paratek: Grant/Research Support

